# CircANXA4 (hsa_circ_0055087) regulates the miR-1256/PRM1 axis to promote tumor progression in colorectal cancer

**DOI:** 10.1016/j.ncrna.2024.03.007

**Published:** 2024-03-13

**Authors:** Guanglan Liu, Xinli Liu, Junfeng Yin, Haijian Zheng, Xinguo Zhu

**Affiliations:** a Department of General Surgery, The First Affiliated Hospital of Soochow University, NO. 188 Shizi Street, Suzhou, 215006, Jiangsu, China; b Department of Digestive Oncology, Cancer Hospital of China Medical University, Liaoning Cancer Hospital & Institute, 44 Xiaoheyan Road, Shenyang, 110042, Liaoning, China; c Department of General Surgery, The Affiliated Hospital of Yangzhou University, NO. 368 Hanjiang Middle Road, Yangzhou, 225000, Jiangsu, China; d Department of Neurology, Ganyu District People's Hospital, No.88 Haicheng Road, Lianyungang, 222100, Jiangsu, China

**Keywords:** Colorectal cancer (CRC), circANXA4, miR-1256, PRM1

## Abstract

Colorectal cancer (CRC) incidence ranks third among malignant cancers with a high propensity for distant metastasis. Despite continuous efforts to improve treatment, the prognosis especially in patients with advanced distant metastasis is low. The mechanism of development and progression of CRC is not fully understood. Non-coding RNAs (ncRNAs) have emerged as essential regulators in cancer progression. Here, we aim to dissect the role of one critical ncRNA, circANXA4, in CRC progression. CircANXA4 expression was analyzed by the GEO database. Differentially expressed circRNAs were identified by the Limma package R software. Expression of circANXA4 and miR-1256 was detected by qRT-PCR. The regulation of circANXA4 on cell proliferation and progression was confirmed with the cell viability assay using cell counting kit-8 (CCK-8) and transwell migration assay. RNA pull-down assay, RNA immunoprecipitation (RIP), and western blot were used to determine the interaction between circANXA4, miR-1256, and protamine1 (PRM1). CircANXA4 was upregulated in both CRC tissues and cell lines. Knockdown of circANXA4 effectively reduced cell proliferation, progression, and migration. Additionally, silencing circANXA4 remarkably increased miR-1256 expression, while reducing PRM1 expression, thereby demonstrating that circANXA4 downregulates miR-1256 expression through a complementary binding site. Rescue experiments revealed the interactions between circANXA4, miR-1256, and PRM1. Pearson correlation analysis revealed that circANXA4 expression positively correlated with PRM1 expression and miR-1256 expression inversely correlated with PRM1 expression. In sum, we demonstrated that circANXA4 promotes cancer cell proliferation and progression by sponging miR-1256 and upregulating PRM1 in CRC.

## 1 Introduction

Colorectal cancer (CRC) incidence ranks third among cancers worldwide, and the incidence rate continues to increase [1]. Despite recent endeavours at treatment, many CRC patients fail to survive the first five years, and the prognosis remains poor, especially for those with advanced distant metastasis [2]. Thus, it would be highly beneficial to identify new therapeutic targets that could be used to prevent CRC metastasis. Moreover, in the era of genomic medicine, elucidating the molecular mechanism provides the opportunity for precise treatment.

CircRNA is a group of non-coding RNAs, which are produced by the non-canonical splicing of precursor RNAs and resist the degradation by exonucleases [3,4]. CircRNAs can bind miRNAs to regulate target gene expression competitively, thus circRNAs have great implications for new biomarkers and therapeutic targets for many cancers, including CRC [[5], [6], [7]]. For example, circCRKL regulates the expression level BCR-ABL by sponging miR-877–5p and has been reported as a potential therapeutic biomolecule to treat chronic myeloid leukemia [8]. CircFOXP1 facilitates CRC progression by inhibiting FOXP1 expression through DNMT1-mediated promotor hypermethylation [9]. CircANXA4 is a relatively new untapped circular RNA, and whether it works in CRC through some molecular mechanism is urgently needed.

MiR-1256 is widely expressed in tumor cells. In gastric cancer cells, it is targeted by circHECTD1, which activates β-catenin/c-Myc pathway to regulate cell activity [10]. Further, miR-1256 was shown to be located on chromosome 12 and is implicated in calving difficulty in Holstein-Friesian dairy cows [11]. Reportedly, miR-1256 and miR-1287 function as tumor suppressors in some cancers [12]. Downregulation of miR-1256 resulted in high tumor growth rates, lymph node metastasis, and shortened overall survival in CRC patients [13]. How these critical cancer-related factors interact to regulate CRC progression is unexplored. Furthermore, protamine1 (PRM1) facilitates G1/S phase transition in CRC cells by activating PI3K/AKT/mTOR pathway [14].

Here, we analyzed circRNA expression in five pairs of CRC patients in the GEO database GSE172229 and found 109 up-regulated and 1004 down-regulated circRNAs. Of these, circANXA4 (hsa_circ_0055087) was highly expressed in CRC tissues, which was correlated with CRC progression. Moreover, circANXA4 expression negatively affected CRC patient prognosis. Mechanically, circANXA4 binds miR-1256 to regulate PRM1 expression, thus contributing to the development and progression of CRC. Therefore, we propose circANXA4 as a potential therapeutic target for CRC treatment.

## 2 Materials and methods

### 2.1 Tissue samples

Tissues dissection was performed at the First Affiliated Hospital of Soochow University, and a total of 70 pairs of cancer and health tissues were collected. The specimens were kept at −80 °C until use.

### 2.2 Bioinformatics analysis

The chip data of circANXA4 expression profiles in cancer and normal adjacent tissues (the accession number GSE172229) were obtained from the Gene Expression Omnibus (GEO). Limma R package (version 3.4.2) was used to construct the heat map presenting differentially expressed circRNAs.

### 2.3 Target gene prediction

The miRNA targets were predicted by circRNA interactome (https://circinteractome.nia.nih.gov/), miRanda (http://www.microrna.org/), and TargetScan (http://www.targetscan.org/),.

### 2.4 RNA isolation and quantitative polymerase chain reaction analysis

The total RNA was isolated using TRIzol reagent (Thermo Fisher Scientific), followed by DNase I treatment and phenol-chloroform extreaction. Gene fragments was amplified using the SYBR Green Quantitative PCR Master Mix (Applied Biosystems, USA). Real-Time qPCR reaction was: initial denaturation at 95 °C for 10 min, followed by 40 cycles of 15 s-long denaturation at 95 °C and 1 min-long elongation at 60 °C. Relative mRNA levels were calculated using the 2^−ΔΔCt^ method. GAPDH and U6 was regarded as internal controls.

### 2.5 RNase R treatment

CircANXA4 circular structure was confirmed by treating CRC cell lines (LOVO and HCT116) with actinomycin D (2 mg/ml). A total of 10 μg RNA was treated by RNase R with incubation for 30 min at 37 °C. Then the circANXA4 and ANXA4 mRNA was measured using qRT-PCR.

### 2.6 Colony formation assay

The CRC cells were grown in DMEM (10% FBS plus) for 2 weeks at 37 °C. Then, the cells were fixed for 15 min in 5 ml of 4% paraformaldehyde, followed by staining with Giemsa (Beyotime) for 30 min. Finally, the colony numbers counted under a light microscope.

### 2.7 Cells and cell culture

The four CRC cell lines (SW620, HT-29, LOVO, HCT116) and the human normal intestinal epithelial cell line (NCM460) were obtained from the American Type Culture Collection (ATCC). The cells were cultured at 37 °C in DMEM (Thermo Fisher Scientific.) plus 10% FBS, 100 U/ml penicillin, and 100 mg/ml streptomycin (Beyotime, China).

### 2.8 Cell transfection

To express circANXA4 in cells, hsa_circ_0055087 was cloned into pLCDH-ciR vector (GenePharma, China). The miR-1256 mimic, small hairpin (sh) RNAs and negative controls were supplied by GenePharma, China. Lipofectamine™3000 reagent (Invitrogen, USA) was used for cell transfection.

### 2.9 Western blot

Radioimmunoprecipitation (RIPA) buffer (Roche Diagnostics, Germany) was applied to lyse cells, and the cell lysates were quantified with BCA Protein Quantification Kit (Bio-Rad, USA). After denature by boiling, the proteins were applied to a 10% sodium dodecyl sulfate (SDS)-polyacrylamide gel. After electrophoresis, the separated proteins were transferred to a polyvinylidene fluoride (PVF) membrane (Millipore, USA) followed by incubation with primary antibodies at 4 °C overnight. After that, horseradish peroxidase-labeled anti-rabbit (ab97051, 1:10000) was used to treat the membrane at room temperature for 1 h. Chemiluminescence detection kit (Beyotime, China) was used to develop the blot.

### 2.10 Cell viability assay

Cell viability assay was performed using cell counting kit-8 (CCK-8, Dojindo Molecular Technologies, China). Briefly, 2 × 10^5^ LOVO or HCT116 cells were transferred into 96-well plates followed by cultivation in an incubator containing CO_2_ (5 %). At 0, 24, 48, and 72 h, 10 μL of CCK-8 solution was applied. Then the cells were further cultured for 4 h at 37 °C, and the optical density at 450 nm was read.

### 2.11 Luciferase reporter gene assay

The psiCHECK system (Thermo Fisher Scientific, USA) was used to perform the luciferase reporter gene assay. The wild-type (WT), mutants of the predicted binding site 1, 2, and 3 of circANXA4, miR-1256, and PRM1 were cloned into a luciferin reporter gene. HEK 293 cells (2 × 10^4^ cells/well) were cultivated overnight in 24 well plates. Then the cells were transfected with WT or mutants of site 1, 2, and 3 as well as miR-1256 mimics (10 nM) or mimics control (10 nM) for 48 h t The Dual-Luciferase Detection Kit (Promega, USA) was adopted to determine the luciferase activity.

### 2.12 RNA immunoprecipitation (RIP)

The cell lysate of LOVO or HCT116 cells was treated with magnetic beads pre-conjugated with immunoglobulin G (1: 500) antibody or Argonaute2 (Ago2; 1: 500). The co-immunoprecipitated RNAs were digested by proteinase K. Finally, the total RNA was extracted and the transcript enrichment was estimated using qRT-PCR.

### 2.13 Transwell migration and matrigel invasion assays

The invasion and migration assays were done using Transwell plates of 8 μm pores (Millipore, USA). The cells (approximately 2 × 10^5^) cells were digested and cultured in 200 μL of medium in the upper chamber without serum. Matrigel was employed for the invasion assays following the manufacturer's protocols (BD Biosciences, USA). After incubation for 24 h at 37 °C, the cells in the lower chamber containing 600 μL medium were collected and fixed with 4% paraformaldehyde followed by staining with 0.1% crystal violet solution (Sigma-Aldrich, USA) for 10 min at room temperature. Invaded and migrated cells in three different fields were quantified and counted under an inverted light microscope (Zeiss, Primovert).

### 2.14 RNA-pull down assay

CircANXA41 and the control probes were custom synthesized by Sangon Biotech (Shanghai). The probes were co-incubated with streptavidin-coated beads for 2 h at 25 °C (Thermo Fisher Scientific, USA). The cell lysates of LOVO or HCT116 cells were incubated with circANXA4 probes overnight at 40 °C. Next, the beads were eluted, and the RNA was collected by TRIzol® reagent (Takara, Beijing, China). QRT-PCR was used to quantify miR-1256 and PRM1.

### 2.15 Statistical analysis

Statistical analysis was performed in GraphPad Prism 7, and data were expressed in mean ± SD. Unpaired student's t-test and one-way ANOVA were used to compare the difference among treatments. The Pearson correlation test was used to assess the relationship between variables. *P* < 0.05 was considered statistically different.

## 3 Results

### 3.1 CircANXA4 upregulation relates to a poor prognosis in CRC

**Fig. 1:**
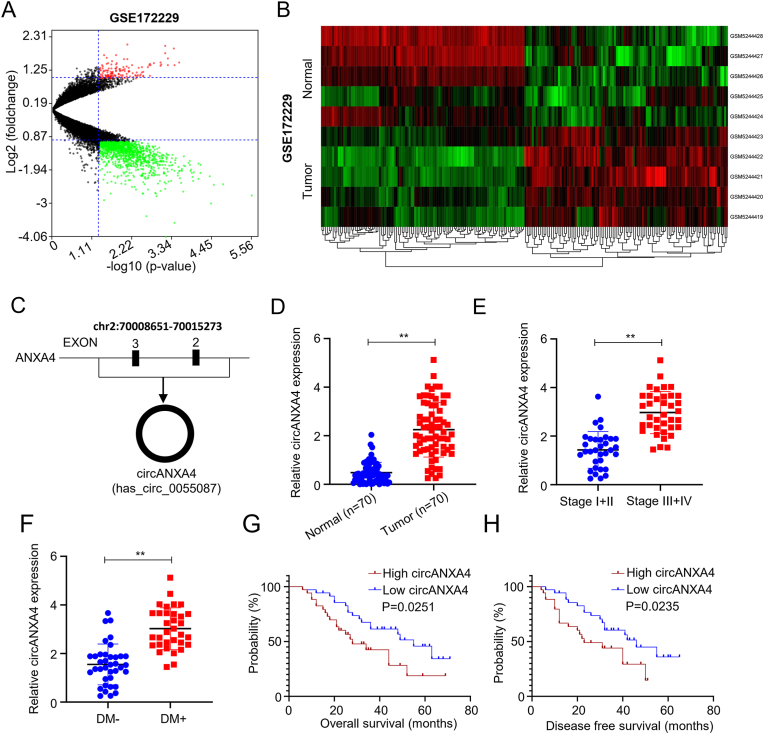
**CircANXA4 expression suggests poor prognosis in CRC.** (A) Volcano plot presented the differentially expressed circRNAs in CRC (CRC) tissues. (B) Limma R package showed the differentially expressed circRNAs in normal and tumor tissues. (C) Schematic of the pre-RNA structure of ANXA4. (D) QRT-PCR analysis indicated that circANXA4 was significantly upregulated in CRC tissues compared to adjacent normal tissues. (E) CircANXA4 expression was correlated with tumor stages. (F) CircANXA4 expression correlates positively with distant metastasis. (G&H) KM-plot showed that circANXA4 expression levels were inversely correlated with the survival rates of patients. The survival curve encapsulates the overall survival and disease-free survival of CRC patients.

To probe circRNA expression in CRC, we downloaded GSE172229 from the GEO database. The volcano plot showed 109 upregulated and 1004 downregulated circRNAs in CRC tissues. Among the upregulated ones, circANXA4 (hsa_circ_0055087) was the most upregulated (*P* < 0.05, Figure A&B). CircANXA4 is located on chromosome 2 (70008651–70015273) and formed by the reverse splicing of exons 2 to 3 of ANXA4 preRNA (Fig. 1C). To further assess the differential expression of circANXA4 in tumor and normal tissues, we used qRT-PCR to quantify circANXA4 in the 70 pairs of cancer and adjacent normal tissues. CircANXA4 was significantly upregulated in the tumour tissue (*P* < 0.05, Fig. 1D). Furthermore, we grouped the 70 CRC patients based on their tumour stages and analyzed the correlation of circANXA4 expression with the tumour stages. The result indicated that circANXA4 expression in patients with advanced CRC stage (stage III + IV) was bigger than those in patients with the earlier stages (stage I + II) (*P* < 0.05, Fig. 1E). Furthermore, we investigated circANXA4 expression based on the metastasis distance in the 70 CRC patients. We divided the patients into two groups, DM- (no distant metastasis) and DM+ (distant metastasis). The result revealed that CRC tissue with distant metastasis exhibited significantly higher levels of circANXA4 expression compared with those with no distant metastasis (*P* < 0.05, Fig. 1F). Based on circANXA4 expression levels, we divided the 70 CRC patients into the high-expression and low-expression groups. Then, we analyzed the relationship between the patient survival rates and circANXA4 expression levels using a Kaplan-Meier-plot. The high circANXA4 expression group exhibited reduced survival rates compared with the low expression group (*P* = 0.0251, Fig. 1G). Additionally, we assessed the disease-free survival of the patients by the Kaplan-Meier method. Patients with high circANXA4 expression levels in CRC tissues exhibited a shorter period of disease-free survival (P = 0.0235, Fig. 1H).

### 3.2 Upregulation of CircANXA4 promotes CRC cell proliferation and metastasis

**Fig. 2:**
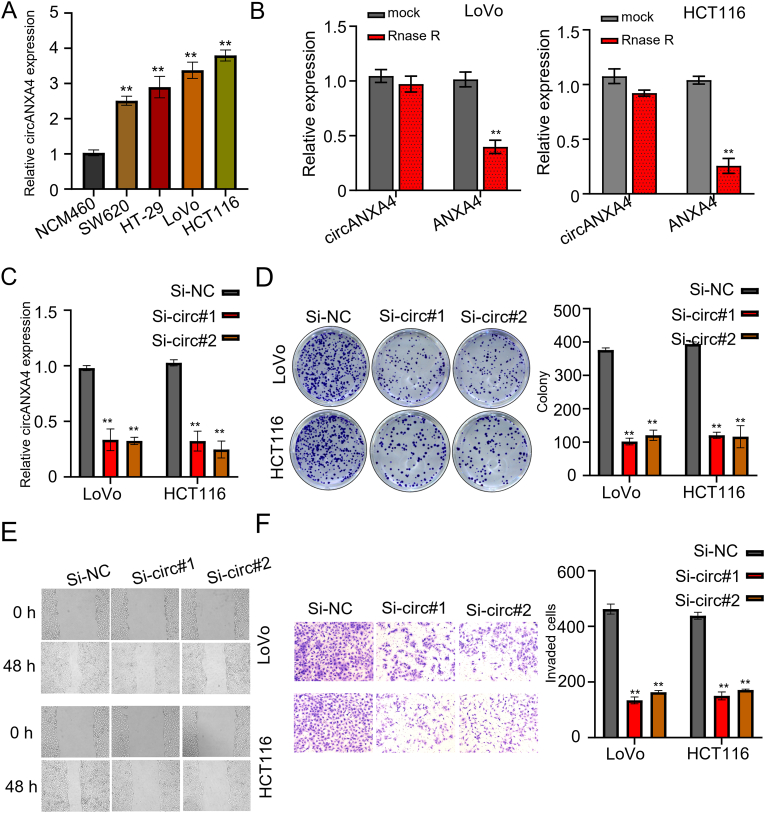
**CircANXA4 expression is upregulated in CRC cell lines and promotes proliferation and metastasis**. (A) CircANXA4 expression levels in four CRC cell lines (SW620, HT-29, LOVO, and HCT116) and the human normal intestinal epithelial cell line (NCM460). (B) QRT-PCR examined the digestion of circANXA4 and linear ANXA4 by RNase. (C) QRT-PCR analysis confirmed that si-circ#1 and si-circ#2 effectively knockdown circANXA4 in HCT116 and LoVo cells compared to the normal control (si-NC). (D) CCK8 assay indicated that knockdown of circANXA4 by si-circ#1 or si-circ#2 effectively reduced the colony-forming ability of HCT116 and LoVo cells. (E) knockdown of circANXA4 significantly reduced the migration ability of HCT116 and LoVo cells. (F) Transwell assay showed that circANXA4 knockdown remarkably reduced the migration ability of HCT116 and LoVo cells.

To study how circANXA4 affects cancer prognosis, we first compared circANXA4 expression between CRC cell lines (SW620, HT-29, LOVO, and HCT116) and the human normal intestinal epithelial cell line, NCM460. CircANXA4 expression was higher in all CRC cell line than in NCM460 (*P* < 0.05, Fig. 2A). Moreover, circANXA4 expression in HCT116 and LoVo cell lines was the highest, and therefore they were used for subsequent experiments (*P* < 0.05, Fig. 2A). Due to the increased stability of circRNA compared to linear RNA and its resistance to degradation [15], we examined the effect of RNase treatment on circANXA4, with linear RNA used as a reference. We extracted linear ANXA4 and circANXA4 from the two cells and then treated them with mock and RNase R. QRT-PCR indicated that circANXA4 was not digested by RNase R, while linear ANXA4 was readily digested by RNase R (*P* < 0.05, Fig. 2B). Furthermore, we designed two siRNAs against circANXA4 and verified the knockdown efficiency by qRT-PCR. Both siRNAs could effectively knock down circANXA4 expression by 50% compared with the normal control (si-NC) in HCT116 and LoVo cells (*P* < 0.05, Fig. 2C). Furthermore, we detected the colony formation ability of the cells with circANXA4 knockdown. The result indicated that circANXA4 knockdown markedly reduced the colony-forming ability in the two cell lines treated with si-circ#1 and si-circ#2 compared with those treated with si-NC (*P* < 0.05, Figure D). Next, scratch test was performed to detect the effects of circANXA4 on the migration of HCT116 and LoVo cells. The result indicated that circANXA4 knockdown could effectively reduce the migration ability of the cell lines (*P* < 0.05, Figure E). Further, transwell assay was performed to determine the effects of circANXA4 on the invasion of HCT116 and LoVo cells. The result indicated that circANXA4 knockdown effectively reduced the invasion capacity of the two CRC cell lines (*P* < 0.05, Fig. 2F).

### 3.3 CircANXA4 sponges miR-1256 to downregulate its expression

**Fig. 3:**
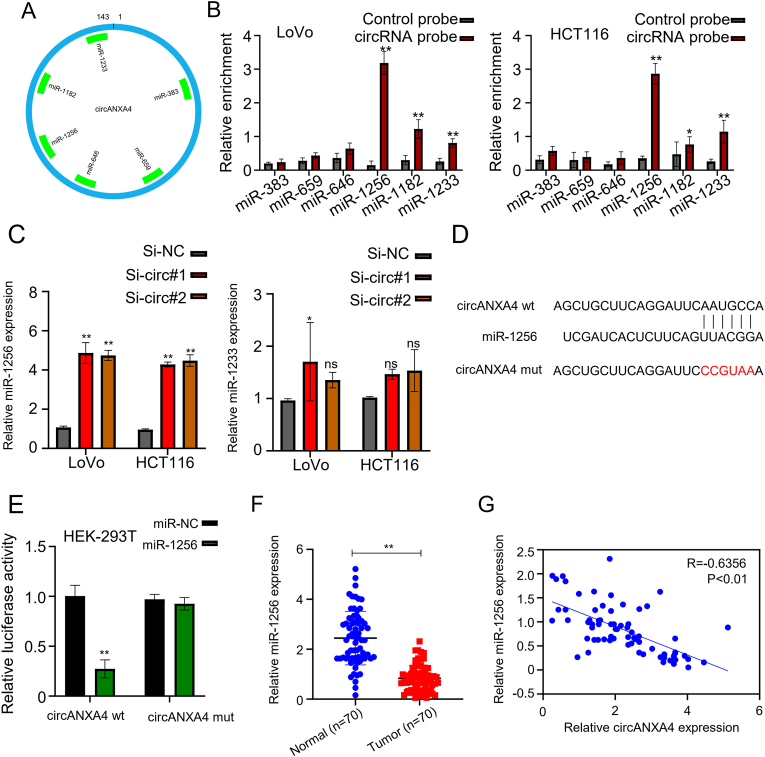
**CircANXA4 acts as a sponge of miR-1256.** (A) We selected the top 6 most common miRNAs based on their conjugation scores, which might indicate potentially functional miRNAs absorbed by circANXA4 in CRC cells. (B) We confirmed the enrichment of circANXA4 and miR-1256 in HCT116 and LoVo cells. (C) QRT-PCR analysis showed that miR-1256 was significantly upregulated when circANXA4 was knocked down by si-circ#1 or si-circ#2. (D) Circular RNA Interactome revealed that circANXA4 has miR-1256 binding site. (E) Luciferase reporter assay revealed that miR-1256 overexpression significantly reduced the relative fluorescence intensity in the HEK-293T cells co-transfected with circANXA4 defective in the three mutated sites. (F) QRT-PCR depicted that miR-1256 was significantly downregulated in CRC tissues compared to normal adjacent tissues. (G) Pearson correlation analysis showed that circANXA4 expression negatively correlated with miR-1256 expression in CRC tissues.

It is well established that circRNAs regulate downstream target gene expression levels by sponging functional miRNAs. To identify the miRNAs targeted by circANXA4, we selected the top six common miRNAs based on conjugation scores (Fig. 3A). We then performed circANXA4 pull-down experiments and checked which miRNAs were associated with circANXA4. QPCR results confirmed that, among the six miRNAs, miR-1256 and miR-1233 were enriched by circANXA4 in both CRC cell lines (*P* < 0.05, Fig. 3B). Further, the sponge of miR-1256 by circANXA4 was confirmed by the observation that knockdown of circANXA4 by si-circ#1 and si-circ#2 markedly upregulated miR-1256 in LoVo and HCT116 cells; however, perhaps the binding ability between miR-1233 and circANXA4 is weak, so it cannot play a regulatory role, resulting in no significant changes in miR-1233 (*P* < 0.05, Fig. 3C). Using circular interactome, we predicted that circANXA4 contains binding site to target miR-1256 (Fig. 3D). To verify this, we made mutants of this site. Then we used the luciferase reporter assay to study the effect of mutations on the interaction between circANXA4 and miR-1256. MiR-1256 significantly reduced the relative fluorescence intensity of wild-type circANXA4, while the extent of fluorescent reduction was less when individual miR-1256 binding site was mutated and the relative fluorescence intensity did not significantly change when all three sites were mutated (*P* < 0.05, Fig. 3E). Moreover, we assessed miR-1256 expression levels in the 70 pairs of cancer and adjacent tissues and found that miR-1256 expression was lower in CRC tissues than in normal adjacent tissues (*P* < 0.05, Fig. 3F). Pearson correlation analysis indicated that the expression levels of circANXA4 were inversely correlated (R = −0.6356, P < 0.01, Fig. 3G).

#### 3.3.1 PRM1 is the target gene of miR-1256

**Fig. 4:**
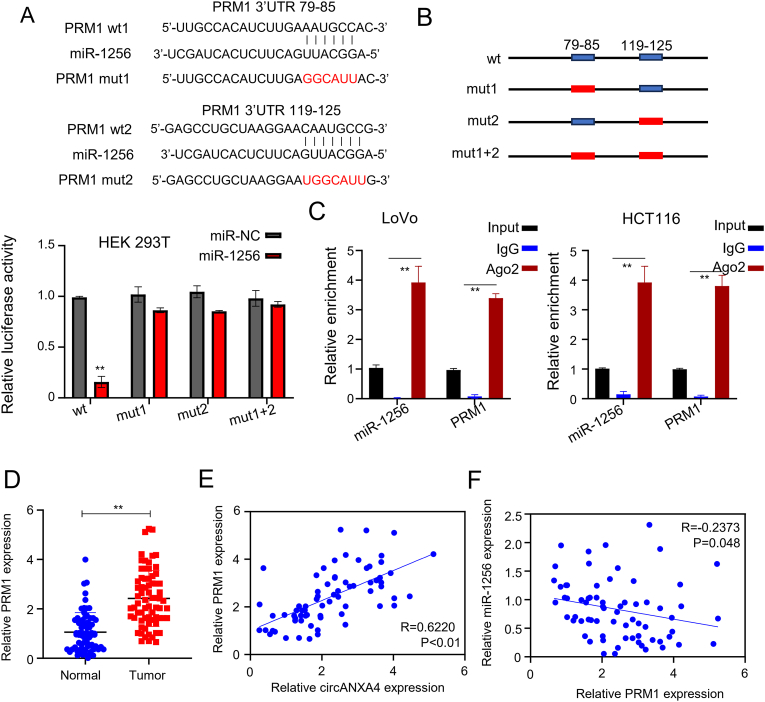
**PRM1 is the target gene of miR-1256.** (A) TargetScan predicted that miR-1256 could bind to the 3′UTR of PRM1. (B) Luciferase reporter assay showed that miR-1256 overexpression significantly reduced the relative fluorescence intensity of HEK-293T cells co-transfected with PRM1 WT but had no significant effect on that of HEK-293T cells co-transfected with PRM1 mutated sequence. (C) RIP-qRT-PCR showed that PRM1 and miR-1256 were enriched by Ago2 but not by IgG. (D) QRT-PCR indicated that PRM1 expression was markedly upregulated in CRC tissues compared to the normal adjacent tissue. (E) Pearson correlation analysis indicated that circANXA4 expression correlates positively with PRM1 expression. (F) PRM1 expression correlates negatively with miR-1256 expression in CRC tissues.

We implemented a target scan to predict the gene targeted by miR-1256 and found that PRM1 could be the target (Fig. 4A). Then, we performed the luciferase reporter assay to compare the interaction of PRM1 wild-type and the mutant to miR-1256. MiR-1256, not miR-NC, significantly inhibited fluorescein expression in the PRM1 wild-type reporter. In contrast, miR-1256 did not significantly affect fluorescein expression in the PRM1 mutant reporter compared with miR-NC mimics in the HEK-293T cells (*P* < 0.05, Fig. 4B). Moreover, we conducted a RIP experiment to determine the recruitment of miR-1256 and PRM1 on anti-Ago2 and anti-IgG in LoVo and HCT116 cells. PRM1 and miR-1256 were efficiently enriched on Ago2 compared to IgG and Normal control (*P* < 0.05, Fig. 4C). Furthermore, we examined PRM1 expression in cancer and adjacent tissues by qRT-PCR. The result revealed that PRM1 expression was remarkably upregulated in the CRC tissues (*P* < 0.05, Fig. 4D). In addition, Pearson correlation analysis indicated that the expression of circANXA4 and PRM1 was positively correlated (Fig. 4E, R = 0.6220, *P* < 0.01), whereas the expression of PRM1 and miR-1256 was inversely correlated (Fig. 4F, R = −0.6356, *P* < 0.048).

### 3.4 CircANXA4 promotes CRC cell proliferation and metastasis through the miR-1256/PRM1 axis

**Fig. 5:**
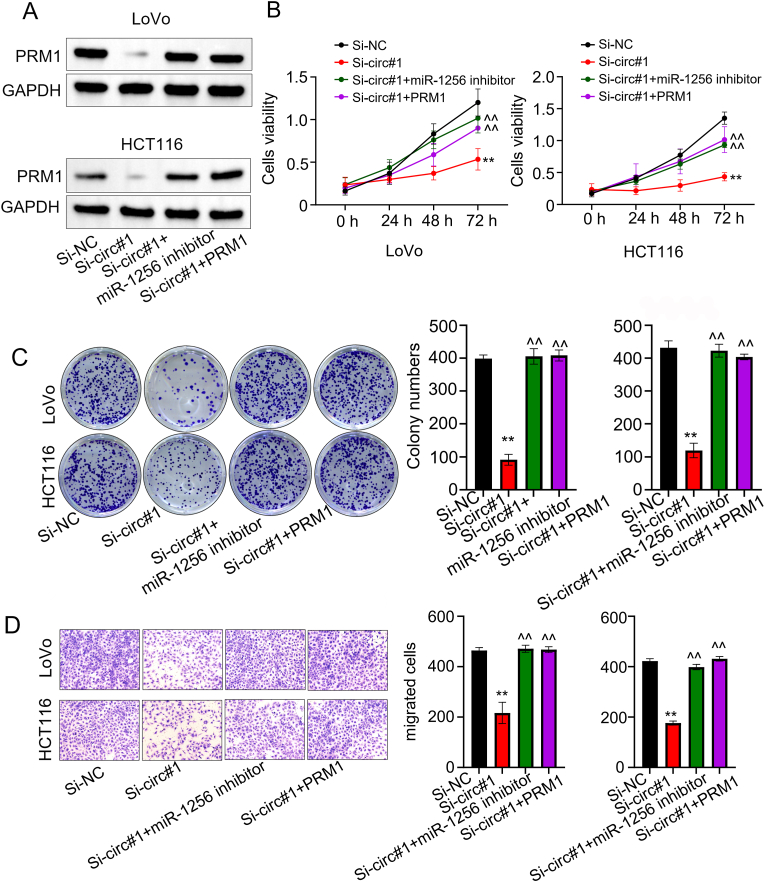
**CircANXA4 promotes the proliferation and metastasis of CRC cells through the miR-1256/PRM1 axis.** (A) Western blot analysis showed that PRM1 protein levels were decreased by circANXA4 knockdown, which was restored by co-transfection with miR-1256 inhibitor. (B) CCK indicated that cell viability was significantly reduced by circANXA4 knockdown in a time-dependent manner, which was reversed by inhibiting miR-1256 or overexpressing PRM1. (C) Colony formation ability of CRC cells was significantly reduced by circANXA4 knockdown, which was rescued by co-transfection with miR-1256 inhibitor and PRM1 overexpression vector. (D) Transwell assay indicated that circANXA4 knockdown significantly decreased the invasion ability of CRC cells, which was reversed by co-transfection with miR-1256 inhibitor and PRM1 overexpression vector.

We performed Western blot to determine PRM1 expression LoVo and HCT116 cells with different gene perturbations. The result revealed that PRM1 expression was remarkably decreased by circANXA4 knockdown and was recovered by co-transfecting either miR-1256 inhibitor or PRM1 overexpression vector (Fig. 5A). Also, we employed CCK8 assay to examine the viability of the cells transfected with si-NC, si-circ#1, si-circ#1+miR-1256 inhibitor or si-circ#1+PRM1 at 0 h, 24 h, 48 h, and 72 h. We found that cell viability was significantly reduced by circANXA4 knockdown compared with normal control. However, the decrease in cell viability caused by circANXA4 knockdown was effectively reversed by co-transfection with miR-1256 inhibitor or PRM1 overexpression vector (*P* < 0.05, Fig. 5B). Further, we used clonogenicity assay to determine colony formation of the cells with the same treatment. We observed that colony formation ability was significantly reduced by circANXA4 knockdown compared to normal control. And this reduction was restored by either miR-1256 inhibitor or PRM1 overexpression (*P* < 0.05, Fig. 5C). Finally, we used transwell experiments to detect the invasive ability of the two cell lines. The result showed that circANXA4 knockdown, not si-NC, significantly inhibited the cell invasion ability. And this inhibition could be reversed by co-transfection of either miR-1256 inhibitor or PRM1 overexpression vector (*P* < 0.05, Fig. 5D).

## 4 Discussion

Cancer poses the worst social, economic, and clinical burden in terms of disability-adjusted life years (DALYs) among all human diseases [16]. CRC ranks second in the cause of cancer mortality and third in occurrence worldwide [17]. Here, we find that circANXA4 is significantly upregulated in CRC cells and promotes cell proliferation and invasiveness, resulting in poor prognosis and high propensity of metastasis.

Differentially expressed profiles of circRNAs between non-cancerous and cancerous tissues as well as between primary and secondary cancer tissues reflect the critical role of circRNAs in cancer progression [17]. Here, we discovered that circANXA4 was markedly upregulated in the CRC tissue compared to the normal adjacent tissue, and that circANXA4 expression was positively correlated with CRC stages and distant metastasis. Additionally, higher levels of circANXA4 expression were closely associated with poor prognosis and low survival time in CRC patients. This result is consistent with a study reporting that circANXA4 facilitates tumor progression and Warburg effects in gallbladder cancer [18]. Collectively, our results confirmed that circANXA4 is abnormally expressed in CRC cells.

CircRNAs have been implicated in cancer development and progression [19]. For instance, circTFF1can regulate lung cancer progression; circ_0000189 enhances glioma cell invasiveness; hsa_circ0001955 overexpression facilitates CRC progression [[20], [21], [22]]. Here, we found that circANXA4 promoted CRC cell proliferation and invasiveness, which is congruent with the fact that circRNAs play a central role in cancer progression.

MicroRNAs and circular RNAs are essential for post-transcriptional regulation [23]. Increasing evidence suggests that miRNAs are sponged by circRNAs, thereby facilitating cancer progression [24,25]. For instance, miR-1256 downregulation has been associated with non-small cell lung cancer [26]. Consistently, we confirmed the upregulation of miR-1256 in CRC tissues. CircANXA4 knockdown upregulated miR-1256. As such, we concluded that miR-1256 is a critical miRNA and sponged by circANXA4 in CRC cells.

We found that PRM1 expression is negatively regulated by miR-1256 in CRC cells. Our data demonstrated that PRM1 was upregulated by circANXA4 while downregulated by miR-1256. Moreover, we observed that PRM1 expression positively correlates with circANXA4 expression, while inversely correlates with miR-1256 expression. This echoes the study reporting that PRM1 is secreted antigen to facilitate G1/S phase transition to respond to nutrient stress in CRC cells [14].

Furthermore, we confirmed the hierarchical regulation of PRM1 by circANXA4 and miR-1256. CircANXA4 knockdown enhanced miR-1256 expression while decreasing PRM1 protein level. Either inhibiting miR-1256 or overexpressing of PRM1 could reverse the effect of circANXA4 knockdown. Knockdown of both circANXA4 and PRM1 reduced CRC cell growth and invasiveness. Although we have demonstrated the critical function of circANXA4 in CRC progression, we believe the regulation of circRNAs in cancer progression is multimodal and complex. For example, hsa_circ_0006732 promoted CRC progression [27], whereashsa_circ_0001666 suppressed CRC cell growth [28]. Therefore, disentanglement of the complexity of circRNA's regulation is key to understanding the etiology of CRC. However, future studies are needed to fully investigate the security of circANXA4 in CRC.

In sum, we delineated a novel regulatory axis of circANXA4/miR-1256/PRM1 involved in CRC progression, Further research on circANXA4 may provide novel insights into CRC diagnosis and treatment, as well as significantly advance therapies in clinical practice.

## Ethics approval and consent to participate

This study was approved by an institutional review board of the First Affiliated Hospital of Soochow University, and written informed consent was obtained from all participants.

## Consent for publication

All patients in this study provided their consent for publication.

## Availability of data and materials

The datasets used and/or analyzed during the current study are available from the corresponding author on reasonable request.

## Funding

The study was funded by Project of Lianyungang Municipal Health Commission (No.202132).

## CRediT authorship contribution statement

**Guanglan Liu:** Writing – original draft, Investigation, Conceptualization. **Xinli Liu:** Conceptualization, Writing – review & editing. **Junfeng Yin:** Investigation, Data curation. **Haijian Zheng:** Writing – review & editing, Conceptualization. **Xinguo Zhu:** Writing – review & editing, Validation, Conceptualization.

## Declaration of competing interest

The authors declare that they have no known competing financial interests or personal relationships that could have appeared to influence the work reported in this paper.
